# Impact of China's Public Hospital Reform on Healthcare Expenditures and Utilization: A Case Study in ZJ Province

**DOI:** 10.1371/journal.pone.0143130

**Published:** 2015-11-20

**Authors:** Hao Zhang, Huimei Hu, Christina Wu, Hai Yu, Hengjin Dong

**Affiliations:** 1 Center for Health Policy Studies, Zhejiang University School of Medicine, Hangzhou, China; 2 Department of Health Information Management, School of Public Health, Hangzhou Medical College, Hangzhou, China; 3 School of Public Health, Zhejiang University School of Medicine, Hangzhou, China; University of Florence, ITALY

## Abstract

**Background:**

High drug costs due to supplier-induced demand (SID) obstruct healthcare accessibility in China. Drug prescriptions can generate markup-related profits, and the low prices of other medical services can lead to labor-force underestimations; therefore, physicians are keen to prescribe drugs rather than services. Thus, in China, a public hospital reform has been instituted to cancel markups and increase service prices.

**Methods:**

A retrospective pre/post-reform study was conducted in ZJ province to assess the impact of the reform on healthcare expenditures and utilization, ultimately to inform policy development and decision-making. The main indicators are healthcare expenditures and utilization.

**Results:**

Post-reform, drug expenditures per visit decreased by 8.2% and 15.36% in outpatient and inpatient care, respectively; service expenditures per visit increased by 23.03% and 27.69% in outpatient and inpatient care, respectively. Drug utilization per visit increased by 5.58% in outpatient care and underwent no significant change in inpatient care. Both were lower than the theoretical drug-utilization level, which may move along the demand curve because of patient-initiated demand (PID); this indicates that SID-promoted drug utilization may decrease. Finally, service utilization per visit increased by 6% in outpatient care and by 13.10% in inpatient care; both were higher than the theoretical level moving along the demand curve, and this indicates that SID-promoted service utilization may increase.

**Conclusion:**

The reform reduces drug-prescription profits by eliminating drug markups; additionally, it compensates for service costs by increasing service prices. Post-reform, the SID of drug prescriptions decreased, which may reduce drug-resource waste. The SID of services increased, with potentially positive and negative effects: accessibility to services may be promoted when physicians provide more services, but the risk of resource waste may also increase. This warrants further research. It is recommended that comprehensive measures that control SID and promote physician enthusiasm be carried out concurrently.

## Introduction

Unreasonably rapid increases in healthcare costs have been a growing problem in many countries worldwide [1]. In China, the healthcare expenditures per visit increased from 987.1 RMB yuan in 2003 to 2,695.1 RMB yuan in 2012, at a growth rate of 10.5% per year [2]; however, between 2003 and 2012, the average income growth rate among Chinese residents was only 9.2% per year in urban areas and 8.1% in rural areas [3]. Within total healthcare expenditures, drug expenditures have been extremely high, representing in 2012 about 51.3% of spending per outpatient episode and 41.3% of spending per inpatient episode [4]. These proportions are among the highest in the world, compared to an average among OECD of around 17% [5]. High drug expenditures constitute a major obstacle to healthcare accessibility in China [6]. Drug expenditures are high on account of a series of factors, including increased production, prices, and utilization [7]. Previous studies have overwhelmingly demonstrated that in China, excessive drug utilization due to supplier-induced demand (SID) is a main contributing factor to the problem of high drug expenditures [8–14].

With SID, the implication is that healthcare suppliers encourage patients to demand a quantity of healthcare production in excess of what they actually need, while taking advantage of an “information gap” [15, 16]. According to the Target Income Hypothesis and Benchmark Model of the Physicians’ Practice, SID is likely to happen when physician income falls below a certain target income (i.e., a certain level of desired income) or when workloads are heavy [17, 18]. The current situation in China makes the system particularly susceptible to SID: in 2010, the average physician wage as a ratio to average wage was 1.09, compared to 1.5–7.5 in most OECD countries [19]. In China in 2012, physicians’ degree of satisfaction with their incomes was about 7.8%, compared to 48% among their American counterparts [20, 21]. Meanwhile, physicians in China suffer from job pressures caused by long work hours and high work intensity, but their income is not proportional to their heavy workload [22, 23].

**Fig 1:**
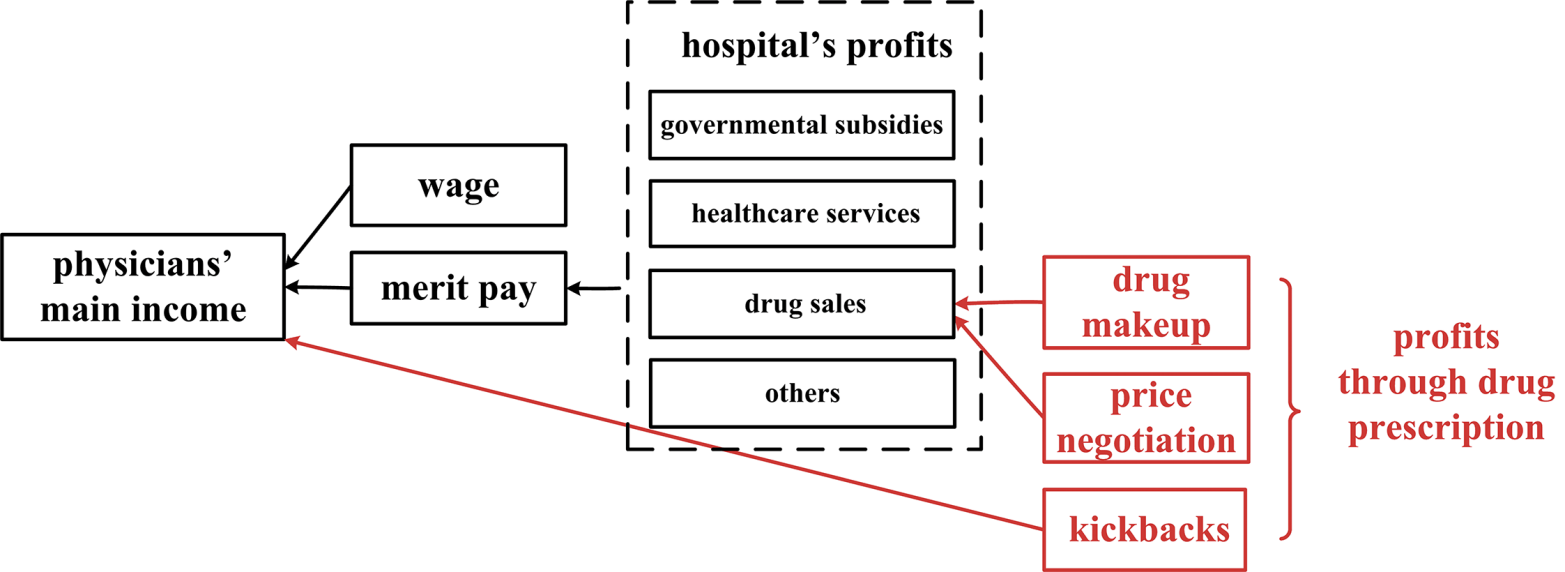
Three main ways to profit from drug prescriptions.

In addition, SID is likely to occur when suppliers are rewarded for increasing healthcare utilization [24]. Among suppliers, public hospitals are representatives of healthcare organizations, while physicians in public hospitals are representatives of medical personnel. Public hospitals are non-profit hospitals owned and funded by the government; as such, these hospitals have a welfare characteristic, and they meet the health demands of the masses [25]. In China, they provide most of the medical care in healthcare delivery—namely, about 90.04% of outpatient care services and 89.03% of inpatient care services in 2012 [2]. Although public hospitals should be funded mainly by the government, since the 1980s health system reform, they have been encouraged to cover their expenses with medical care profits, given the government’s financial strain [26]; therefore, the actual government funding has decreased sharply, from about 35% of the total hospitals’ income in the 1970s to less than 10% in the 2010s [2, 27]. Today, the economic operation of hospitals relies heavily on healthcare income: in 2012, healthcare income, government funding, and other forms of income accounted for 89.46%, 8.16%, and 2.38% of total income, respectively [2]. Among healthcare income, drug and service incomes accounted for 40.07% and 29.65% of hospitals’ total income, respectively [2]. Drug income may generate profits for hospitals profits through drug markup and price negotiation; markups are authorized by the government, while negotiations are irregular and “under the table” [28–30] (Fig 1). The drug markup policy was instated in the early 1950s, to compensate for hospital expenditures [29]. The markup is 15–30% of the purchase price, according to relevant policies; thus, it may generate for hospitals about 5–9% of their total income [31]. Drug price negotiation is conducted between hospitals and pharmaceutical companies in secret, with the drug purchase price being discounted and the gap between the actual purchase price and the official purchase price being returned to the hospitals. The hospitals manage to escape government surveillance, because the drug purchase quantity is not fixed by the government, and so an actual contract stating an actual purchase quantity and discounted purchase price can be signed as an ostensible contract with a lower purchase quantity and an official purchase price [30, 32]. In these ways, both drug markup and price negotiations can generate profits for hospitals.

Physicians may also generate profits through drug sales. Physicians’ incomes comprise a fixed wage, merit pay, and other allowances [20]. Merit pay can have a great influence on physicians’ medical behavior, since it is related to medical performance and constitutes a large portion of their income (e.g., 45.1% in 2013) [20, 33]. Merit pay also relates to hospital profits: according to China’s current income distribution system, merit pay is distributed to medical departments and then to physicians, based on their healthcare and economic outcomes [34]. Since drug sales can generate profits for hospitals, drug prescriptions can also increase merit pay for physicians. In addition, physicians may also receive under-the-table kickbacks from pharmaceutical companies or sales personnel [35, 36]. In this way, both the hospitals and the physicians are rewarded for drug sales, and increasing the quantity of drug prescriptions written may therefore generate higher profits. Under such circumstance, SID is more likely to occur.

Meanwhile, the low prices of healthcare services aggravate the aforementioned problem. The prices of healthcare services (i.e., all healthcare services except drug prescriptions) are rather low; for 90% of the services, the fees charged are lower than their actual costs [37]. The cost of physician labor is seriously underestimated, and physician income cannot be adequately compensated through service fees [38]. Therefore, physicians are keen to generate profits through drug prescriptions, rather than by providing services. As a result, the low enthusiasm among physicians in providing services is also an obstacle to an accessible healthcare system in China.

China’s government is devoted to promoting a well-functioning healthcare delivery system, which has undergone three main stages of development. The first stage occurred during China’s planned economy period, after the liberation and before the Reform and Opening Up (1949–78). The hospitals were funded and managed almost completely by the government. This model may have allocated resources reasonably in times of resource scarcity, and it was held up as a model for developing countries in providing universal healthcare [39]. The second stage occurred after the Reform and Opening Up in 1978, when market-oriented economic reforms were launched. A corresponding health system reform was implemented in the early 1980s, which gave autonomy to hospitals that lacked public finance funding [39]. Hospitals were encouraged to cover their expenses with medical care profits, as mentioned; however, the health system reform of the 1980s created problems, with the welfare nature of the public hospitals being diluted by profit-seeking. Furthermore, system performance was low, owing to inefficiency, inequality, and high expenditures [40]. Facing these challenges, a new round of health system reforms were implemented in 2009 [41, 42]; these aimed to ensure that basic healthcare services worked for the good of the public by distributing healthcare resources reasonably and promoting healthcare accessibility, thus re-emphasizing the role of the government in the healthcare delivery system [42]. Five major tasks were launched, including a pilot reform of public hospitals [43, 44]. This public hospital reform aimed to recover the public hospitals’ welfare nature, by increasing government investment, separating healthcare behavior from supplier profits, and compensating appropriately for service costs [41]. Government funding supports the system mainly in the form of investments in medical insurance; this pushes suppliers to provide services for financial compensation [45]. Two measures are being implemented directly at the public hospital level: (1) eliminating the markup on Western drugs—which constitutes about 15% of the purchase price—to separate drug prescriptions from supplier profits, and (2) increasing the prices of medical services, in order to compensate to some extent for the low service costs. However, the growth rate of the prices was capped, so that the increased prices may compensate for 90% of the profit loss created by the decreasing drug prices [46].

County-level hospitals in ZJ province were selected as the sites of the reform pilot, because county-level hospitals are at the interface of the urban and rural medical systems and thus provide healthcare services to 70% of the population in the county area [47]; additionally, ZJ province is representative of China’s socioeconomically developed regions, which have relatively complete health delivery systems and a stable social environment. ZJ province is located in the southern portion of the Yangtze River Delta in east China. In 2013, its gross domestic product (GDP) was 3.7568 trillion RMB yuan, ranking fourth in the country; its health expenditures represented 4.45% of its GDP. Its permanent population as of 2013 was 54.43 million; its number of (assistant) registered physicians, nurses, and hospital beds per 1,000 people was 2.52, 2.41, and 4.18, respectively [48, 49].

**Fig 2:**
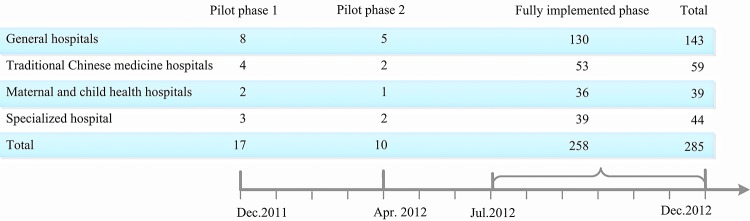
Time schedule for county-level hospital reform in ZJ province.

The reform was launched in December 2011. By the end of 2012, all 285 county-level hospitals in ZJ province had implemented the reform, including 143 general hospitals, 59 traditional Chinese medicine hospitals, 39 maternal and child health hospitals, and 44 specialized hospitals. Among the 143 general hospitals, 13 hospitals launched the reform as a pilot in pilot phases 1 and 2 (December 2011–April 2012); the remaining 130 began the reform in its fully implemented phase (July–December 2012) [46] (Fig 2).

To assess the effects of the public hospital reform on healthcare expenditures and utilization, we compared data from county-level hospitals in ZJ province before and after the reform; our endpoint in doing so is to help better inform policy development and decision-making.

## Methods

### Study design and sampling

We conducted a retrospective pre/post-reform case study in December 2013 of county-level hospitals in ZJ province. General hospitals were chosen, so as to preclude bias relating to various medical specialties. In all, 130 hospitals that had instituted the reform since July 2012 were involved in the study, to maintain a consistent starting time with regard to the reform. These hospitals implemented the reform measures, and correspondingly adjusted their internal financial systems and administer regulations during the July–December 2012 period; therefore, the data in this period were unstable and not representative. We selected data from the January–June 2013 period as the post-reform data, and the average data of January–June 2011 and January–June 2012 as the baseline pre-reform data. Since the pre/post-reform data were from the same month, seasonal variation in health demands were avoided.

### Data collection

We modeled our questionnaire after the questionnaire pertaining to the public hospital reform survey, which had been conducted by the Ministry of Health in the pilot region (Health Office Medical Care Administration File [2010] 1034) (S1 Table). Our questionnaire collected information pertaining to hospital size, staffing, financing, aggregated patient characteristics, and healthcare expenditures per visit. Health policy research experts, health administration officers, and hospital administrators were consulted and asked to review the instrument, and modifications were subsequently made as needed.

The questionnaires were then distributed to the hospitals through the Health and Family Planning Commission (HFPC) of ZJ province (the former Province Health Bureau); they were executed by the information department of each hospital. Each questionnaire was reviewed by the researchers for errors, inconsistencies, and omissions, and data were recaptured as necessary. This process was supervised by the HFPC, which guaranteed the authenticity of the data and ensured the timely gathering of completed questionnaires.

These data were also submitted by the hospitals to the HFPC of ZJ province in official financial and performance reports, and then publicly released following summary and analysis (http://www.zjwst.gov.cn/col/col320/). However, the publicly released data were annual or quarterly aggregated data, which were not suitable for this study; therefore, we used questionnaires to collect the data that our study required.

### Hypothesis

It is assumed that the elimination of drug markup may reduce suppliers’ drug-prescription profits and further reduce SID in this field; therefore, SID-related drug utilization could also decrease. Increasing service prices may compensate to some extent for the low service costs, make physicians more enthusiastic about providing services, and alleviate profit-seeking through drug prescriptions; for these reasons, SID-related drug utilization may also decrease. However, increasing service prices may generate more profits for the hospitals, further increase the merit pay of physicians, and consequently increase SID-related service utilization.

### Indicators

#### Drug expenditures and utilization

A main indicator used in this study is drug expenditures per visit (DEPV). In calculating drug utilization, the World Health Organization recommends the use of particular metrics; these include the defined daily dose (DDD), the prescribed daily dose (PDD), and metrics based on drug cost. The first two metrics are not adopted in our study. DDD is the assumed average maintenance dose per day for a drug; however, in reality, many drugs are prescribed on the basis of individual characteristics and pharmacokinetic considerations, rather than DDD [7]. PDD is the actual average dose prescribed of a drug, but because in this study we found that many drugs lacking an Anatomical Therapeutic Chemical Classification are prescribed, such a calculation is difficult [50]. However, metrics based on drug cost are suitable for use in an overall analysis; their use is especially appropriate in this study, since the data we obtained were aggregated data pertaining to all drugs in the hospitals, rather than a single drug. Cost is the product of price and utilization (i.e., cost = price × utilization); thus, utilization = cost / price. In this study, the indicator of utilization is drug utilization per visit (DUPV), and the indicator of cost is DEPV; DUPV = DEPV / drug price. The DEPV can be calculated, but in this study, the price cannot be directly obtained; therefore, the absolute value of DUPV cannot be calculated. However, there is a relationship between drug prices in the pre/post-reform periods: drug price pre-reform = (1 + 15%) × drug price post-reform, as the post-reform drug price is the drug purchase price, and the pre-reform drug price is the purchase price plus drug markup. Thus, the relative number of changes in DUPV (ΔDUPV) can be calculated. The metric we adopt in this study is ΔDUPV (Formula 1), where P represents drug price, and the subscript “pre” and “post” indicate initial pre-reform data and final post-reform data, respectively.

$$\Delta \text{DUPV}\%=\frac{{\text{DUPV}}_{\text{post}}{-\text{DUPV}}_{\text{pre}}}{{\text{DUPV}}_{\text{pre}}}\times 100\%=\frac{{\text{DEPV}}_{\text{post}}/{\text{P}}_{\text{post}}-{\text{DEPV}}_{\text{pre}}/{\text{P}}_{\text{pre}}}{{\text{DEPV}}_{\text{pre}}/{\text{P}}_{\text{pre}}}\times 100\%$$ (Formula 1)

#### Service expenditures and utilization

Service expenditures per visit (SEPV) is also adopted in this study. The change in service utilization is calculated using Formula 2, employing the same calculation method for drug utilization. SUPV represents service utilization per visit, and P represents service price.

$$\Delta \text{SUPV}\%=\frac{{\text{SUPV}}_{\text{post}}{-\;\text{SUPV}}_{\text{pre}}}{{\text{SUPV}}_{\text{pre}}}\times 100\%=\frac{{\text{SEPV}}_{\text{post}}/{\text{P}}_{\text{post}}-{\text{SEPV}}_{\text{pre}}/{\text{P}}_{\text{pre}}}{{\text{SEPV}}_{\text{pre}}/{\text{P}}_{\text{pre}}}\times 100\%$$ (Formula 2)

In addition, the relationship between pre- and post-reform service prices is calculated via Formula 3, according to policy measures wherein the service-price growth rate is capped so that it may compensate for 90% of the profit loss created by the reduced drug price [46].

$$\left|{\text{DEVP}}_{\text{pre}}\;\times \;\Delta {\text{P}}_{\text{drug}}\;\times \;90\%\right|=\left|{\text{SEPV}}_{\text{pre}}\;\times \;\Delta {\text{P}}_{\text{service}}\right|$$ (Formula 3)

#### Hospital income composition

We also adopt hospital income composition, since hospitals’ reliance on healthcare income to fund economic operations is a factor that induces profit-seeking among suppliers. The indicators include the proportion of government funding, drug income, and service income in total income.

In all comparisons, economic data are adjusted by an inflation rate as follows: 2012 unit prices are adjusted to 2011 prices by deflating them by 1.3% [51], and 2013 unit prices to 2011 prices by deflating them by 1.6039% [52].

### Statistical analysis

A paired t-test was used to test the pre-post differences in the indicators before and after the reform. These tests are executed with the use of SAS Version 9.2 (SAS Institute, Cary, NC, USA). A post-hoc power analysis is conducted via GPower 3.1(Franz Faul, Uni Kiel, Germany) to assess whether the data are adequate for analysis.

### Demand curves analysis

It is difficult to calculate the health demands created by SID, because the change in demand may also have been caused by patient-initiated demand (PID), according to the Law of Demand–Price; therefore, we use demand curves to illustrate SID-promoted utilization [15]. The curves are drawn in Microsoft Visio 2010, with price along the vertical (y) axis and quantity along the horizontal (x) axis; thus, the curves form an inverse demand function. The slope of the demand curve is dP/dQ, where P represents price and Q represents quantity; the elasticity coefficient is (dQ/dP) P/Q. The points on the curves represent the demand quantities at a certain price [15]. In this study, the prices are drug or service prices, and the demand quantities are drug or service utilization. Although precise price and utilization data cannot be obtained, the relationship between the pre- and post-reform data can be obtained; therefore, the demand curves can be drawn on the axes with scale marks that represent the magnitude relationship. The changes in demand created by PID may theoretically move along the demand curve pre-reform, while the actual demand influenced by the reform may move along the demand curve post-reform. We draw the pre/post-reform demand curves on the same axis, compare them, and understand that the difference between them may be caused by SID.

## Results

All 130 general county-level hospitals executed the questionnaire; however, 38 questionnaires were improperly executed, leaving 92 questionnaires for analysis; as such, the effective response rate was 70.7%. Although the effective response rate was not high, there were no statistically significant differences in the general information between complete cases and all 130 hospitals, including the number of beds (p = 0.063), the number of staff members (p = 0.076), the number of outpatient care visits (p = 0.086), the number of inpatient care visits (p = 0.093) (S2 Table). In addition, a post-hoc power analysis was conducted, with all the power values (π) exceeding 0.95 (S3 Table). Although there are no formal standards for power, most researchers assess the power of their tests using π = 0.80 as a standard for adequacy [53]; therefore, the data from the hospitals that had fully completed the questionnaire were deemed adequate for analysis.

### Hospital income composition

The income composition of hospitals changed post-reform. Healthcare income still constituted the majority of total income, but the percentage of healthcare income in total income decreased, from 81.63% pre-reform to 79.78% post-reform. The percentage of drug income in total income decreased, while the percentage of service income in total income increased; these changes were caused mainly by increased service income, rather than reduced drug income. The number of visits jumped from a median of 193,388 per year to 215,304 in outpatient care, and from 5,828 to 6,707 in inpatient care. This led to 30.12% of the increase in service income in outpatient care and 32.12% of the increase in service income in inpatient care; the remaining 69.88% of the increased service income in outpatient care and 67.88% of the increased service income in inpatient care may have been caused by increased SEPV. In addition, the government funding underwent no significant change, and the percentage of government funding in total income decreased (Table 1).

**Table 1:** Hospital income composition.

Indicators	Mean ± SD (Million RMB yuan)			Income composition (%)(mean of each component / mean of hospitals’ total income)	
	Pre-reform	Post-reform	t	Pre-reform	Post-reform
Hospitals’ total income	111.61 ±103.31	133.41 ±127.89	-6.294**	100	100
Government financial funding	4.86 ± 6.38	5.00 ± 6.00	-0.309	4.35	3.75
Drug income in outpatient care	23.54 ± 21.91	23.57 ± 22.66	-0.077	21.09	17.67
Drug income in inpatient care	25.69 ± 26.32	24.85 ± 26.22	1.772	23.02	18.63
Service income in outpatient care	17.92 ± 16.48	23.33 ± 20.91	-9.594**	16.05	17.49
Service income in inpatient care	23.96 ± 25.27	34.68 ± 36.69	-8.527**	21.46	25.99
Others	15.65 ± 18.29	21.97 ± 24.66	-3.441**	14.02	16.47

Note: A t-test was used to compare DEPV pre- and post-reform

**p < 0.01.

### Drug expenditures and utilization

The drug price decreased by 13.04% after the reform. Compared to the pre-reform data, DEPV significantly decreased in both outpatient and inpatient care (emergency treatment also included) (Tables 2 and 3). However, DUPV did not decrease: the DUPV of outpatient care increased (p < 0.1), and that of inpatient care underwent no significant change (p = 0.198) (Table 3). This indicates that the decrease in DEPV was created by price reductions, rather than by DUPV.

**Table 2:** Drug expenditures per visit.

Indicators	Mean ± SD (RMB yuan)	
	Pre-reform	Post-reform
Drugs in outpatient care	80.32 ± 22.81	74.42 ± 24.35
Drugs in inpatient care	2892.66 ± 1107.78	2471.18 ± 906.89

**Table 3:** Drug expenditures per visit (adjusted by inflation rate) and changes in drug utilization per visit.

Indicators	DEPV adjusted by inflation rate				
	Mean ± SD pre-reform (RMB yuan) (a)	Mean ± SD post-reform (RMB yuan) (b)	t	Growth rate (%)	ΔDUPV (%)(c) $\text{c}=\frac{\text{b}/1-\text{a}/\left(1+15\%\right)}{\text{a}/\left(1+15\%\right)}\times 100\%$
Drugs in outpatient care	79.79 ± 22.66	73.25 ± 23.97	3.778**	-8.20	5.58
Drugs in inpatient care	2873.46 ± 1100.65	2432.17 ± 892.57	7.519**	-15.36	-2.66

Note: DEPV represents drug expenditures per visit; DUPV represents drug utilization per visit. A t-test was used to compare DEPV pre- and post-reform (adjusted by inflation rate)

**p < 0.01.

**Fig 3:**
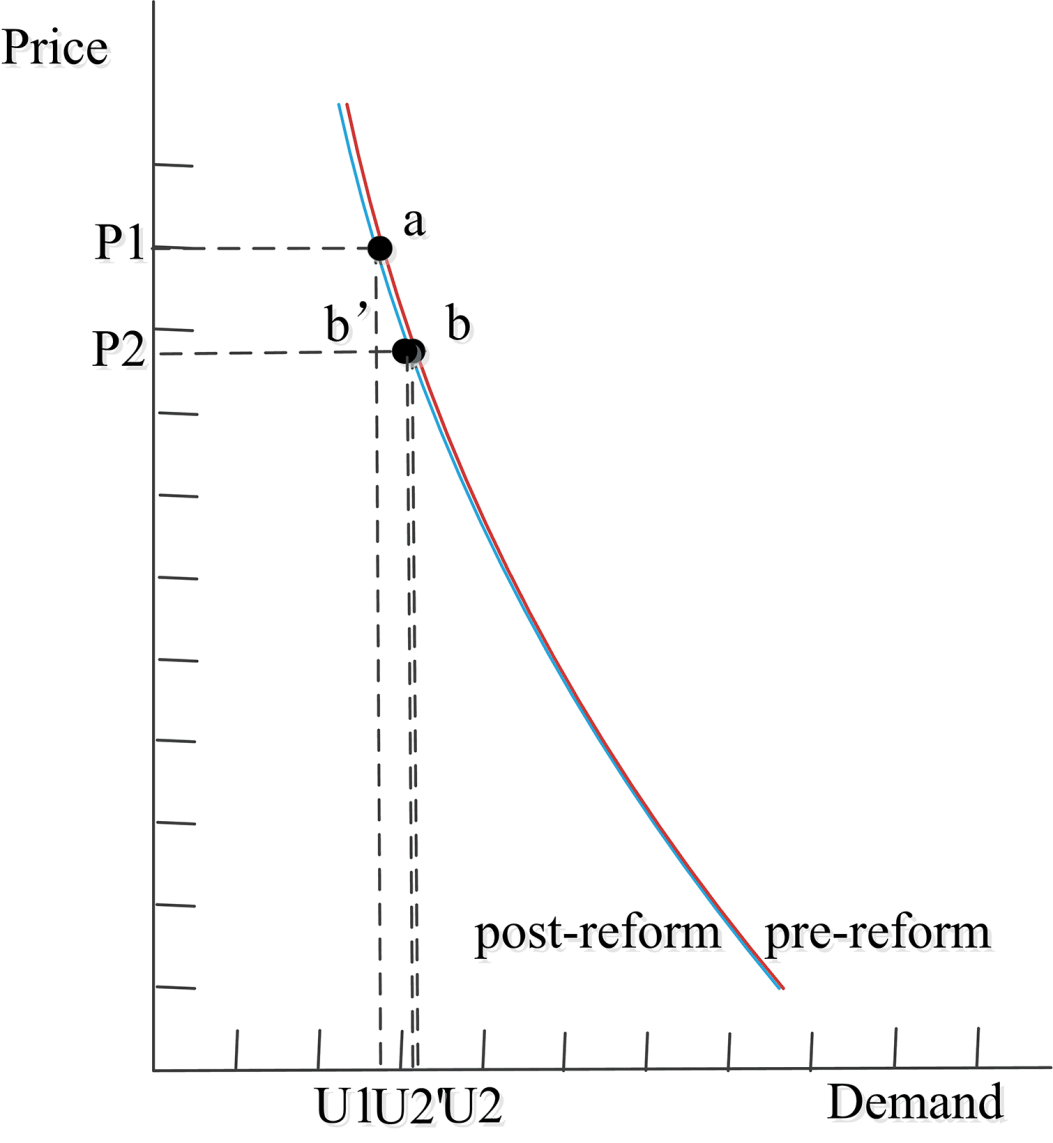
Demand curve of drugs in outpatient care, pre/post-reform. Note: P1 and P2 represent drug prices pre- and post-reform; U1 represents drug demand pre-reform, U2 represents theoretical drug demand post-reform, and U2' represents actual drug demand post-reform.

**Fig 4:**
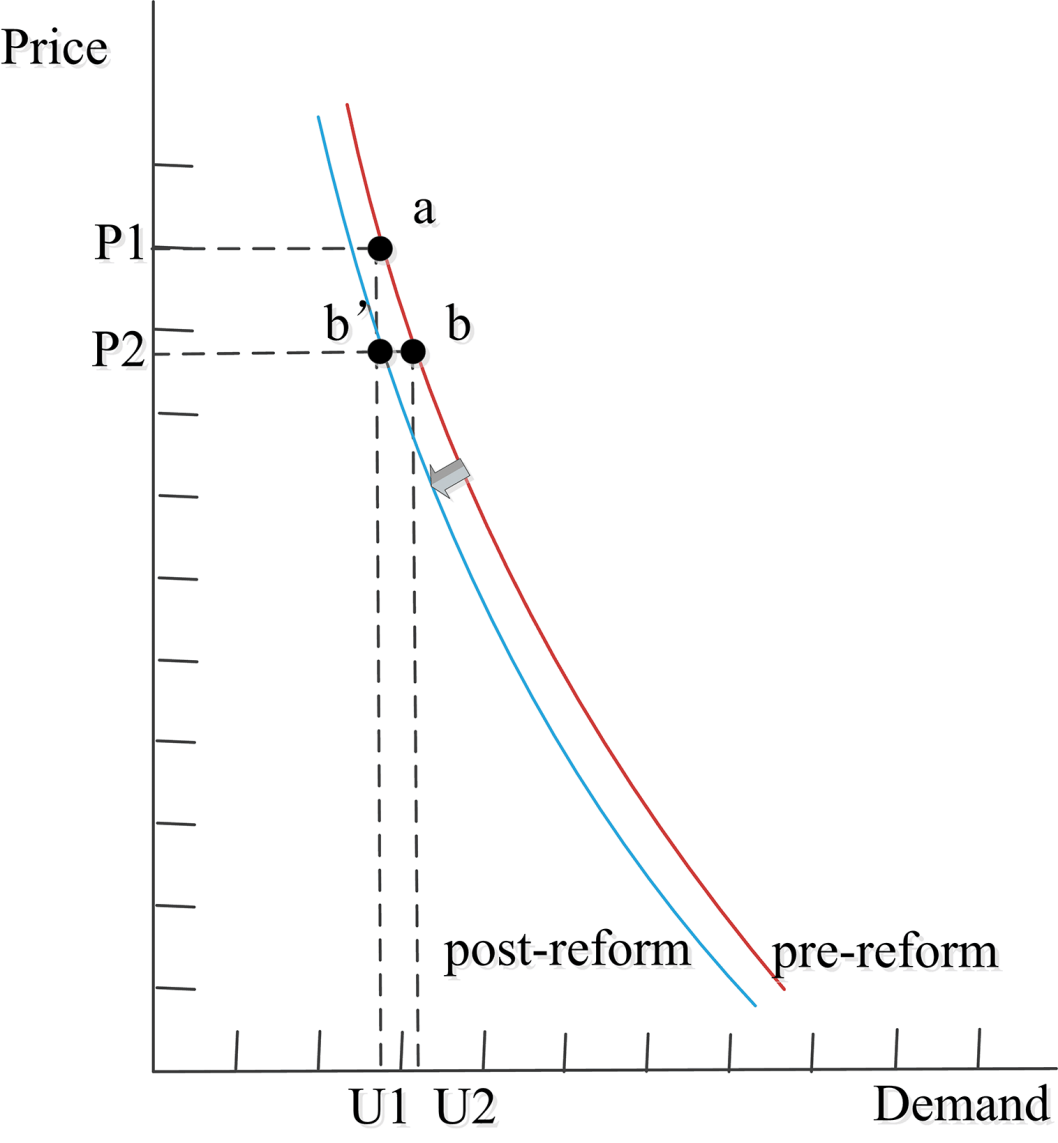
Demand curve of drugs in inpatient care, pre/post-reform. Note: P1 and P2 represent drug prices pre- and post-reform; U1 represents drug demand pre-reform, U2 represents theoretical drug demand post-reform, and U1 also represents actual drug demand post-reform.

The demand curve for inpatient-care drugs shifted after the reform. According to National Health Services Survey data, the price elasticity coefficient of demand is –0.49 in outpatient care and –0.47 in inpatient care [54]. In theory, when drug prices decrease by 13.04%, the drug demand created by PID may increase by 6.38% and 6.13% in outpatient and inpatient care, respectively. The equilibrium point may move from a to b (Figs 3 and 4); in reality, however, the equilibrium point moved from a to b', with drug demand increasing by 5.58% in outpatient care and remaining unchanged in inpatient care. This indicates that the actual drug demand influenced by the reform was lower than the theoretical one. In addition, such a trend is more obvious in inpatient care than in outpatient care.

### Service expenditures and utilization

The price of services increased by 16.08% and 12.90% in outpatient and inpatient care, respectively. Compared to pre-reform conditions, SEPV increased in both outpatient and in inpatient care (Tables 4 and 5). SUPV also increased in outpatient care, with the increased SUPV care causing 26.05% of the increased SEPV. In inpatient care, the increased SUPV caused 47.31% of the increased SEPV. The remainder of the increased SEPV (i.e., 73.95% in outpatient care and 52.69% in inpatient care) was caused by increased prices.

**Table 4:** Service expenditures per visit.

Indicators	Mean ± SD (RMB yuan)	
	Pre-reform	Post-reform
Services in outpatient care	59.56 ± 17.23	73.33 ± 16.14
Services in inpatient care	2632.50 ± 867.52	3393.01 ± 1231.99

**Table 5:** Service expenditures per visit (adjusted by inflation rate) and changes in service utilization per visit.

Indicators	SEPV adjusted by inflation rate				
	Mean ± SD pre-reform (RMB yuan) (a)	Mean ± SD post-reform (RMB yuan) (b)	t	Growth rate (%)	ΔSUPV (%) (c) $\text{c}=\frac{\text{b}/{\text{P}}_{\text{post}}-\text{a}/{\text{P}}_{\text{pre}}}{\text{a}/{\text{P}}_{\text{pre}}}\times 100\%$
Services in outpatient care	58.66 ± 14.92	72.17 ± 15.88	-13.833**	23.03	6.00
Services in inpatient care	2615.21 ± 862.05	3339.45 ± 1212.54	-9.804**	27.69	13.10

Note: SEPV represents service expenditures per visit; SUPV represents service utilization per visit. A t-test was used to compare SEPV pre- and post-reform (adjusted by inflation rate)

**p < 0.01.

**Fig 5:**
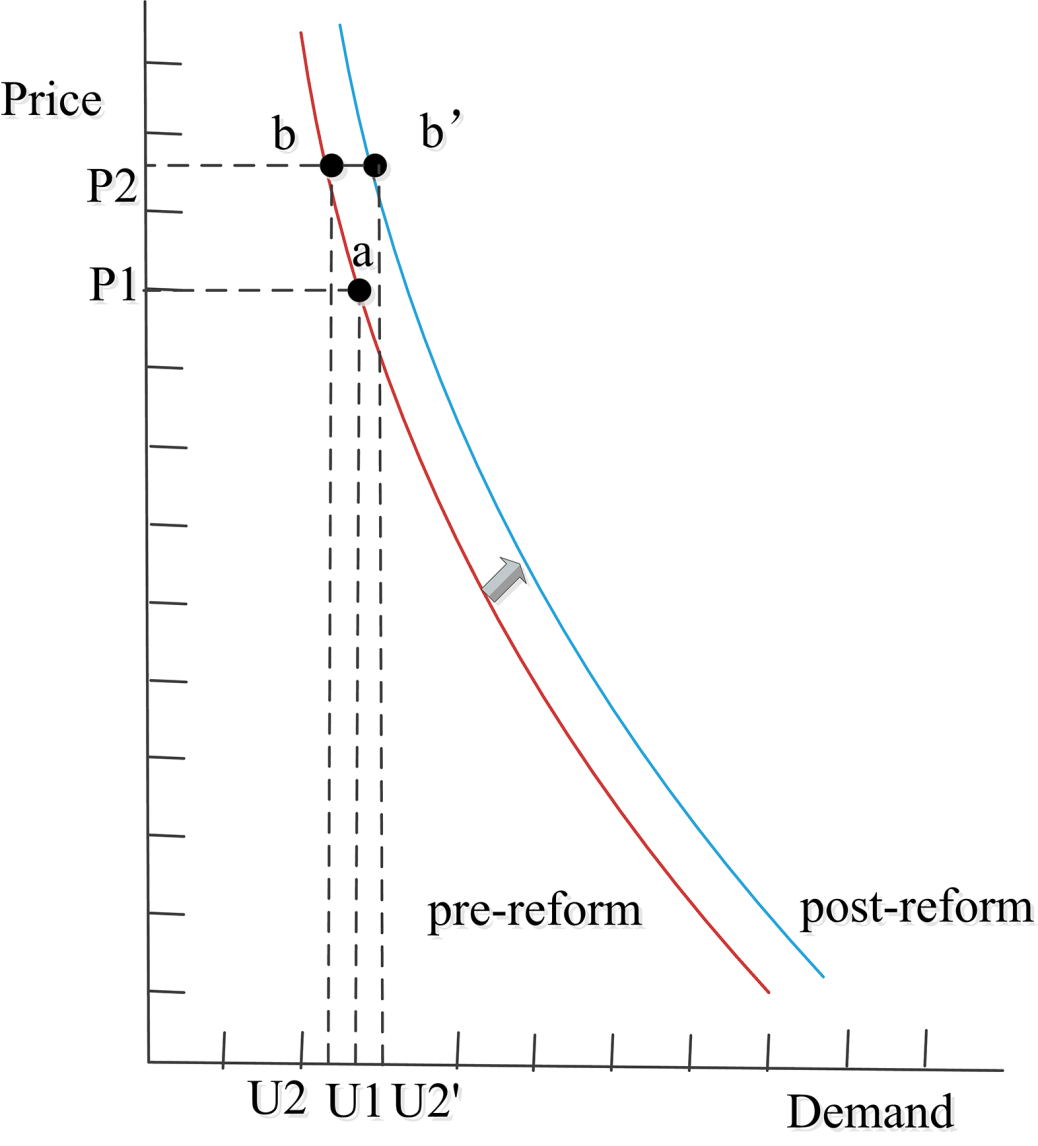
Demand curve of services in outpatient care, pre/post-reform. Note: P1 and P2 represent service prices pre- and post-reform; U1 represents service demand pre-reform, U2 represents theoretical service demand post-reform, and U2' represents actual service demand post-reform.

**Fig 6:**
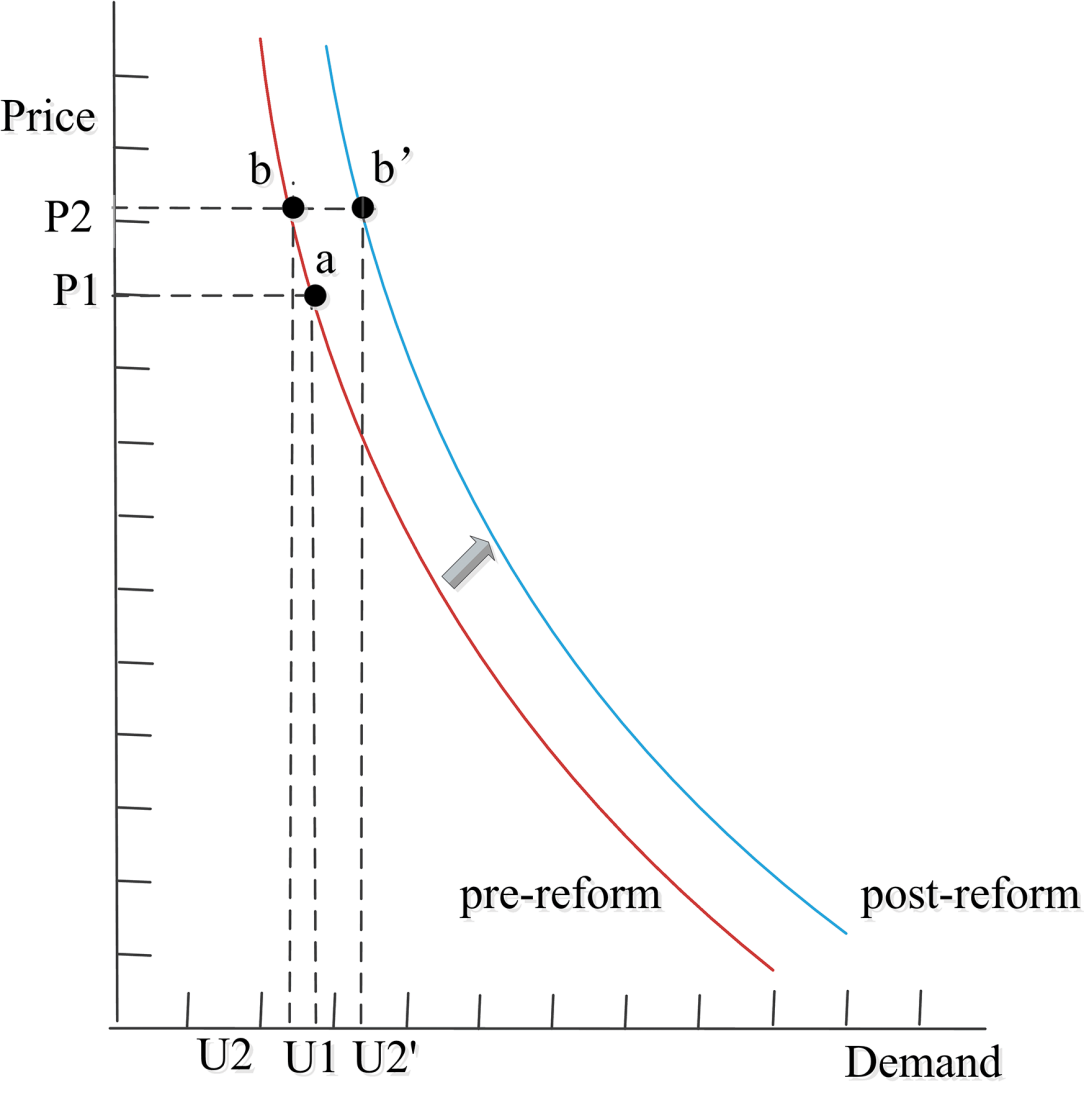
Demand curve of services in inpatient care, pre/post-reform. Note: P1 and P2 represent the service prices pre- and post-reform; U1 represents service demand pre-reform, U2 represents theoretical service demand post-reform, and U2' represents actual service demand post-reform.

The demand curve of services shifted (Figs 5 and 6). According to the aforementioned price elasticity coefficients [47], in theory, when service prices increase by 16.08% in outpatient care and by 12.90% in inpatient care, the service demand for outpatient and inpatient care may decrease by 7.87% and 6.01%, respectively, and the equilibrium point may move from a to b. In reality, the equilibrium point is b' (services demand increased by 6.00% in outpatient care and by 13.10% in inpatient care). This indicates that the actual service demand influenced by the reform was lower than the theoretical one.

## Discussion

This study assesses the impact of public hospital reform policy on healthcare expenditures and utilization in China. The findings indicate that, post-reform, the DEPV decreased; however, this decrease was caused by price reduction, rather than a decline in DUPV. The SEPV increased, and this was caused both by increased service prices and increased SUPV. These results are consistent with those of previous studies that draw the conclusion that in ZJ province, the public hospital reform may have reduced DEPV while increasing SEPV. Those previous studies include one we conducted in 22 pilot county-level hospitals [55], and another that was conducted in three pilot county-level hospitals [56]. Since the previous studies were conducted in hospitals that instituted the reform in pilot phases 1 and 2 (December 2011–April 2012), the sample size is small and its representativeness is therefore limited; however, this study was conducted in more than 70% of the hospitals that had carried out the reform in the fully implemented phase—the representativeness of which is much higher. In addition, this study also assesses the change in healthcare utilization, which may more directly reflect changes in medical care behavior. Thus, this study is an important supplement to and extension of the existing literature.

Medical utilization is influenced by several factors, including price and non-price determinants. A change in the price, *ceteris paribus*, will result in a change in medical utilization created by PID. Such changes will not lead to a shift in the demand curve. However, non-price determinants can cause a shift in the demand curve: these non-price determinants mainly include consumer income, the price of replacements, insurance, preferences, and SID [8]. In this study, the non-price determinant is likely to be SID, since the reform measures may influence physician profits and further influence physician behavior. Our findings may provide evidence of SID in healthcare, and support those studies that argue the existence of SID in this field [24, 57]. The actual amount of drug utilization influenced by the reform was lower than that predicted by theory; this indicates that drug utilization caused by SID could have decreased after the reform. The actual service utilization increased while that predicted by theory may decrease; this in turn indicates that service utilization created by SID may increase. Medical care utilization may increase or decrease when the level of SID increases or decreases (in the same direction) [17, 18], and so it can be deduced that the level of SID in drug prescriptions decreases while the level of SID in services increases. Although exact data pertaining to SID-driven utilization cannot be obtained, such change trends can be certain.

These results are consistent with our hypothesis, and the following conclusions can be drawn. The reform reduces drug-prescription profits by eliminating drug markups; additionally, it compensates for service costs by increasing service prices. Post-reform, the SID of drug prescriptions decreased; however, SID in services increased, since higher service prices may have generated more profits for the hospitals and further increased the merit pay of physicians.

Such changes in medical care behavior may have complex effects. Reduced SID with respect to drug prescriptions may reduce the waste of medical resources, lead to reasonable drug utilization, and promote healthcare accessibility [58]. The increased SID in services may have both positive and negative effects, since it may promote healthcare accessibility in low-resource settings, but waste healthcare resources in resource-rich settings [8]. Pre-reform, low enthusiasm on the part of physicians to provide services was an obstacle to healthcare service accessibility; when service prices increase, the value of the physician labor force is promoted, and the physicians may be encouraged to provide more services. Nevertheless, there may also be the potential to waste resources, and this warrants further research. In addition, increased service prices may also lead to a decrease in PID, which is unfavorable for healthcare service accessibility [1].

In addition, unlike that in inpatient care, the shift in the drug demand curve in outpatient care is insensitive to policy measures. There are some plausible explanations for this. There may have been less SID in outpatient care than in inpatient care pre-reform, since the medical insurance reimbursement rate in outpatient care was lower than that of inpatient care [59]. In outpatient care, physicians may show less SID behavior, given the relatively higher proportion of out-of-pocket payments; therefore, utilization may have been less influenced by the reform. Another explanation is that there is less demand for services in outpatient care than in inpatient care [4], and the financial loss caused by drug price reduction is unlikely to be compensated by increased service income; therefore, physicians may remain keen to derive drug-related profits through price negotiations or kickbacks.

The reform policy measures would not change physicians’ medical care behavior thoroughly, since the underlying motivation of SID would not have changed post-reform. Low incomes and heavy workloads among physicians remain unchanged by this reform, and so the physicians’ target income level may still be unreached; this may strongly motivate SID. Besides, profits still exist in drug prescriptions—mainly through price negotiations and kickbacks. Drug price negotiations and kickbacks have been focal points of society; however, they are difficult to control, given their clandestine nature.

The reform aims to recover the welfare nature of public hospitals by re-emphasizing the role of the government in the healthcare delivery system; however, governments in ZJ province do not play a critical role in financial subsidies for hospitals. The government funding remains at low levels, and hospitals still rely on healthcare profits; therefore, the motivation for SID may increase. The ZJ province government has invested more than 60 billion RMB yuan in New Rural Cooperative Medical System and Urban Residents Basic Health Insurance to subsidize demanders [60]; however, it has both positive and negative effects, since it may encourage suppliers to supply more services for compensation, but increase SID in services. It is necessary to find a balance between encouraging supplier enthusiasm and controlling SID, and this is a topic that requires further study.

There is a limitation inherent in the physicians’ salary-incentive mechanism. Merit pay, which is typical performance-related pay, is intended to promote the cost-effectiveness of human resources. It should be paid based on skill and performance [61]; however, skill and performance is difficult to evaluate, and staff members’ financial outcomes are usually adopted instead [62]. Therefore, physicians are likely to try to maximize utility and ignore healthcare quality. Besides, use of the fee-for-services (FFS) payment model—the main medical insurance payment model in China—may also aggravate this problem [63]. A growing body of evidence demonstrates that with FFS payment models, medical resources could be overused, since the fees are paid by a third party; as such, physicians may increase healthcare utilization to derive profits, without heeding cost controls [24].

In addition, in China there is a lack of a competitive and supervisory mechanism that could otherwise effectively control SID. Market competition in China’s healthcare delivery system is inadequate, since public hospitals absolutely predominate in terms of the number of agencies, the number of patient visits, human resources, and the like [64–66, 4]; thus, the number of patients that public hospitals take in remains steady, regardless of medical services quality. Under such conditions, physicians may ignore healthcare quality in pursuit of profits [67]. Meanwhile, professional ethics and practice norms may provide internal discipline and external constraints with respect to physician practice [68, 69], but the system still needs a reputation-based mechanism that promotes professional ethics; it also requires a comprehensive set of clinical practice guidelines by which standard practice norms can be set.

Since the reform addressed only a few of the aforementioned underlying reasons, it cannot effectively promote ideal physician behavior. It has been suggested that measures that target more of the problematic factors be carried out concurrently, including increases to physician income and reductions to their workloads; increases to government funding, to reduce profit-seeking among suppliers; adjustments to physicians’ salary-incentive mechanisms; changes to health insurance payment models; and establishing competitive and supervisory mechanisms, *inter alia*.

There are several limitations inherent in this research. There is a lack of control group comprising hospitals that did not implement the reform, as all county-level hospitals in ZJ province had carried out the reform by December 2012. Instead, we adopted pre- and post-reform observations, as there had been no significant changes in policy or social environment that could have influenced these hospitals. We adjusted the financial data by applying an inflation rate; however, the price fluctuations set by the market do not adequately reflect in the calculations, since the drug market is a relatively steady one [70]. Caution should be taken in generalizing findings from general county-level public hospitals in ZJ province to other county-level public hospitals in China. Differences in socioeconomic, regulatory, and administrative contexts may influence the effect of policy implementation, and so further study is needed in which the sample size is expanded. Additionally, healthcare expenditures and utilization are only two aspects that reflect physician behavior; more research is needed on the many other aspects that relate to reasonable healthcare behavior, including antibiotics utilization and intravenous injection utilization, among others.

## Conclusion

The introduction of China’s public hospital reform policy seems to have had a comprehensive effect on healthcare expenditures and utilization there. The drug expenditures per visit decreased mainly on account of reduced prices. The service expenditures per visit increased, because of increases to both prices and utilization. The shift in the drug demand curves provides evidence of SID in healthcare behavior. The actual level of drug utilization influenced by SID is lower than that predicted by theory, and this indicates that SID with respect to drug prescription has decreased. The actual level of service utilization influenced by SID is higher than that predicted by theory, and this indicates that SID with respect to services has increased. Therefore, the elimination of drug markup and increases to service prices may decrease SID in drug prescriptions and increase SID in services. This study is an important supplement to and extension of previous studies that assessed the influence of public hospital reform on county-level hospitals, and it thus supports studies that argue the existence of SID in medical care.

The decrease in drug expenditures per visit may promote drug accessibility, and the increase in service expenditures per visit may reasonably compensate for service costs, to some extent. The decrease in SID with respect to drugs may reduce the waste of medical resources, and promote reasonable drug utilization; the increase in SID with respect to services, on the other hand, may have both positive and negative effects, since it may encourage physicians to be more enthusiastic about providing services (i.e., by promoting labor-force value), but it may also waste healthcare resources. These matters warrant further research. China’s reform policy measures could not attain ideal behavior changes among physicians, since many of the underlying factors of that behavior have remained unaddressed. We recommend that comprehensive measures that look to control underlying SID factors and promote physician enthusiasm be carried out concurrently.

## Supporting Information

S1 Table Questionnaires on public hospital reform in Zhejiang province. (DOCX)

S2 Table The representative of the 92 hospitals. (DOC)

S3 Table Post-hoc power analysis. (DOC)
