# Supplementary material for: Impact of China's Public Hospital Reform on Healthcare Expenditures and Utilization: A Case Study in ZJ Province
Source: PLoS One. 2015 Nov 20;10(11):e0143130. doi: 10.1371/journal.pone.0143130 (PMC4654516; doi:10.1371/journal.pone.0143130)
Supplement: S1 Table — (DOCX) [file pone.0143130.s001.docx]

| **S1 Questionnaires of public hospital reform in Zhejiang Province**  The questionnaire was modeled after the *questionnaire of public hospital reform survey conducted by in pilot region* (Health Office Medical Care Administration File [2010] 1034) | | | | | |
| --- | --- | --- | --- | --- | --- |
| **Hospital code** |  | | | |  |
| **Hospital Name** |  | | | |  |
| **The time when the reform began** |  | | | |  |
| **Indicators** | | **time** | | | |
|  |  | 2011.1-2011.6 | 2012.1-2012.6 | 2013.1-2013.6 | |
| **General information** | |  |  |  | |
| Numbers of beds | |  |  |  | |
| Number of staff | |  |  |  | |
| Number of physicians | |  |  |  | |
| Number of nurses | |  |  |  | |
|  | | | | | |
| **Financial information** | |  |  |  | |
| 1. Total income | |  |  |  | |
| 1.1 Government subsidies | |  |  |  | |
| 1.2 Healthcare income | |  |  |  | |
| 1.2.1 Inpatient care income | |  |  |  | |
| 1.2.1.1 Drug income | |  |  |  | |
| Western drug | |  |  |  | |
| Chinese traditional drug | |  |  |  | |
| 1.2.1.2 Service income | |  |  |  | |
| Register income | |  |  |  | |
| Examine income | |  |  |  | |
| Treatment income | |  |  |  | |
| Surgery income | |  |  |  | |
| Assay income | |  |  |  | |
| Material income | |  |  |  | |
| Others | |  |  |  | |
| 1.2.2 Outpatient care income | |  |  |  | |
| 1.2.2.1 Drug income | |  |  |  | |
| Western drug | |  |  |  | |
| Chinese traditional drug | |  |  |  | |
| 1.2.2.2 Service income | |  |  |  | |
| Beds income | |  |  |  | |
| Examine income | |  |  |  | |
| Treatment income | |  |  |  | |
| Surgery income | |  |  |  | |
| Assay income | |  |  |  | |
| Material income | |  |  |  | |
| Nursing income | |  |  |  | |
| Others | |  |  |  | |
| 1.3 Others | |  |  |  | |
|  | |  |  |  | |
| 2. Hospital expenditure | |  |  |  | |
| 2.1 Service expenditure | |  |  |  | |
| 2.2 Drug expenditure | |  |  |  | |
| 2.3 Government subsidies expenditure | |  |  |  | |
| 2.4 Others | |  |  |  | |
|  | | | | | |
| **volume of service** | |  |  |  | |
| 1. outpatient visit(including emergency visit) | |  |  |  | |
| 2. inpatient visit | |  |  |  | |

**Contact information _____________ date_____________**

**Checking information______________ date_____________**
