# Supplementary material for: Impact of China's Public Hospital Reform on Healthcare Expenditures and Utilization: A Case Study in ZJ Province
Source: PLoS One. 2015 Nov 20;10(11):e0143130. doi: 10.1371/journal.pone.0143130 (PMC4654516; doi:10.1371/journal.pone.0143130)
Supplement: S2 Table — (DOC) [file pone.0143130.s002.doc]

**S2 The representativeness of the 92 hospital**

**Normal distribution test**

| general information | Kolmogorov-Smirnov Z | P |
| --- | --- | --- |
| number of beds (92 hospitals) | 1.663 | 0.008 |
| number of beds (130 hospitals) | 1.707 | 0.006 |
| number of staff (92 hospitals) | 1.473 | 0.026 |
| number of staff (130 hospitals) | 1.712 | 0.006 |
| number of outpatient care visits (92 hospitals) | 1.745 | 0.005 |
| number of outpatient care visits (130 hospitals) | 1.587 | 0.013 |
| number of inpatient care visits (92 hospitals) | 1.556 | 0.016 |
| number of inpatient care visits (130 hospitals) | 1.689 | 0.007 |

Note：Kolmogorov-Smirnov test was used to test the normal distribution of the general information of 92 hospitals and 130 hospitals. The general information includes the number of beds, staff, and visits.

Homogeneity of variance test

| general information | Levene statistics | P |
| --- | --- | --- |
| number of beds | 0.003 | 0.957 |
| number of staff | 0.948 | 0.331 |
| number of outpatient care visits | 3.687 | 0.056 |
| number of inpatient care visits | 1.436 | 0.232 |

Note：Levene test was used to test the normal distribution of the general information of 92 hospitals and 130 hospitals. The general information includes the number of beds, staff, visits.

One-sample Wilcoxon signed rank test of the general information of 92 hospitals

|  | median | | P |
| --- | --- | --- | --- |
| general information | test median(130 hospitals) | 92 hospitals |
| number of beds | 382 | 379 | 0.063 |
| number of staff | 531 | 521 | 0.076 |
| number of outpatient care visits | 197,111 | 176,042 | 0.086 |
| number of inpatient care visits | 5,325 | 5,048 | 0.093 |

Note: One-sample Wilcoxon signed rank test was used to compare the median of the general information of 92 hospitals to the test median (the median of 130 hospitals).
