# Supplementary material for: Impact of China's Public Hospital Reform on Healthcare Expenditures and Utilization: A Case Study in ZJ Province
Source: PLoS One. 2015 Nov 20;10(11):e0143130. doi: 10.1371/journal.pone.0143130 (PMC4654516; doi:10.1371/journal.pone.0143130)
Supplement: S3 Table — (DOC) [file pone.0143130.s003.doc]

**S3 Table Post-hoc power analysis**

**Post-hoc power analysis of T-test for healthcare expenditure per visit adjusted by inflation rate (α=0.05,n=92)**

| Indicators | Mean of difference | SD of difference | Power |
| --- | --- | --- | --- |
| drug expenditure per outpatient visit | 6.273 | 15.838 | 0.983 |
| drug expenditure per inpatient visit | 441.286 | 562.933 | 1.000 |
| services expenditure per outpatient visit | -13.755 | 9.381 | 1.000 |
| services expenditure per inpatient visit | -724.237 | 708.531 | 1.000 |
| healthcare expenditure per outpatient visit | -12.613 | 26.214 | 0.998 |
| healthcare expenditure per inpatient visit | -490.991 | 1300.578 | 0.974 |

**Post-hoc power analysis of Wilcoxon signed rank test for hospital income composition (α=0.05,n=92)**

| Indicators | Mean of difference | SD of difference | Power |
| --- | --- | --- | --- |
| governmental financial subsidies | 0.006 | 0.045 | 1.000 |
| drug income in outpatient care | 0.038 | 0.041 | 1.000 |
| drug income in inpatient care | 0.040 | 0.035 | 1.000 |
| healthcare services income in outpatient care | -0.018 | 0.040 | 1.000 |
| healthcare services income in inpatient care | -0.042 | 0.023 | 1.000 |
